# Epidemiological, serological, and genetic evidence of influenza D virus infection in humans: Is it a justifiable cause for concern?

**DOI:** 10.1080/21505594.2022.2150443

**Published:** 2023-01-04

**Authors:** Widaliz Vega-Rodriguez, Hinh Ly

**Affiliations:** Department of Veterinary & Biomedical Sciences, College of Veterinary Medicine, University of Minnesota, Twin Cities, MN, USA

**Keywords:** Influenza viruses, influenza A, influenza B, influenza C, influenza D, zoonotic infection

Several new human viruses have emerged or re-emerged in recent years (e.g., SARS-CoV-2 [1–5], monkeypox virus [6], and Langya henipavirus LayV [7]) that have made significant global and/or local public health impacts. Yet, there are other viruses that appear to be spreading rapidly among livestock in recent years [8–10]. These newly emerged viruses can pose a significant economic burden on the agricultural sector and possibly to human health if they are left unchecked. The focus of this article will be on IDV, as mounting evidence has suggested that it has the potential to establish a recurring infection cycle in human populations, similar to that of influenza A (IAV) and influenza B (IBV) viruses, and might therefore require a seasonal immunization program, if it is proven necessary.

IDV was discovered in Oklahoma, USA in 2011 in an ailing pig [11]. Since then, it has been epidemiologically and serologically identified in cattle with respiratory disease [12]. Cattle appears to be the primary reservoir [13] for virus amplification and possible transmissions into other animal species [14,15], including swine, small ruminants, and camelids. Experimentally, IDV can infect and/or be transmitted to ferrets, mice, guinea pigs, cattle, and of course, cattle and pigs [11,13,16,17]. The ability of this virus to infect and/or transmit to multiple animal species raises a real concern about its zoonotic potential. Here, we summarize several lines of evidence that implicate IDV infection of humans and how to combat it.

A serological survey of sera collected from the general human population in the United States and Canada in 2011 showed a 1.3% seroprevalence of IDV in 4 out of 316 tested individuals [11] (Figure 1). A related and more comprehensive study in Italy found a relatively sharp increase in seroprevalence for IDV in its general human population from 5.1% in 2005 to 46% in 2014 [18]. Both studies linked serological evidence to environmental and occupational exposure to IDV. Since IDV has been identified in swine and cattle, it is important to assess the potential and degree of seroprevalence of workers in swine and cattle farms. It is remarkable that a very high level of seroprevalence (almost 100%, 34/35 IDV antibody-positive sera) was found among cattle workers in the United States [19]. However, a much lower degree of seroprevalence (4.9%, 4/82 IDV antibody-positive sera) was detected among swine veterinarians in Italy [20]. Regardless, these studies suggest that humans could be exposed to IDV under different settings (Figure 1), assuming that the potential for cross reactivity to other viruses (e.g., influenza C virus or ICV) could be ruled out, and highlight the significance of a potential for zoonotic transmission of IDV. However, it is important to note that, despite the serological evidence, there is no direct evidence that IDV can infect humans.

**Figure 1.**
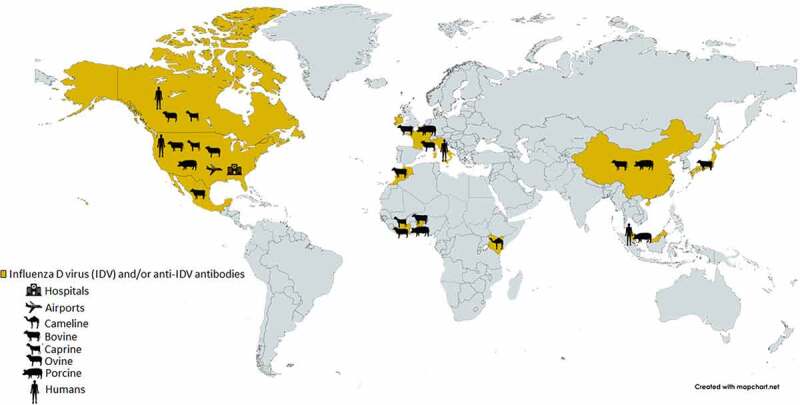
Locations where IDV or IDV antibodies have been detected, as well as the locations of environmental/occupational exposures are shown. Adapted from (14).

The closest and perhaps strongest evidence for a direct human infection by IDV came from a study, which showed that the IDV genomic materials could be detected in a nasal wash sample collected from a swine farm worker in Malaysia [21] (Figure 1). Additionally, IDV genomic materials have also been detected in aerosol samples collected from public settings, such as in an international airport [22], and in a hospital emergency room [23] in the United States. Collectively, these are corroborating evidence for zoonotic potential of IDV, which has the potential to pose a significant public health problem, like in the case of SARS-CoV-2 transmissions in public and/or congregate settings, including school, entertainment, hospice care and hospital settings, etc. [24–28]. Yet, it is important to note that, thus far, neither the complete viral sequence has been detected in those samples nor a replication-competent IDV has been isolated from them.

Experimentally, it has been shown that IDV can infect a variety of human cell types, including the human alveolar epithelial A549 cells, rectal tumor HRT-18 G cells [29,30], and other human primary well-differentiated airway epithelial cells [30], as well as animal cells, including the Madin-Darby canine kidney MDCK cells, swine testicle ST cells, and the African green monkey kidney Marc-145 cells [11]. The ability of IDV to infect a variety of human and animal cells appear to be dependent on its ability to attach to and enter the cells via a common cellular receptor known as 9-O-acetyl-N-acetylneuraminic acid [29]. Additionally, the unique conformational structure of the IDV haemagglutinin esterase fusion (HEF) glycoprotein can partly explain its broad cellular tropism [29]. It is noteworthy that IDV has been shown to be able to experimentally infect and/or transmit to ferrets, mice, guinea pigs, and of course, pigs, all of which have been used as surrogate models of human influenza virus infection [11,13,16,17]. These findings highlight a real potential of IDV to infect and/or maintain not only in humans but also in other reservoirs, such as wild and domestic animals. As many of these animals have interacted with humans, it is important to perform routine serological surveys of individuals, such as swine and cattle workers, for evidence of IDV infections.

While IDV has not been shown to cause a significant form of illness in humans and therefore should not be of a significant public health threat, it has the potential to adapt and evolve (via spontaneous mutagenesis) in any of the susceptible hosts into a potentially more virulent form due to its error-prone RNA-dependent RNA polymerase that cannot faithfully copy its genome [31]. This has important public health implications in that some of the IDV natural variants might be able to establish its foothold in either the general or certain human population(s), such as the young or senior and/or immunocompromised individuals, and therefore could continue to evolve into different viral variant forms that might complicate the surveillance effort and/or might require seasonal immunization program, if an IDV vaccine is determined to be necessary and can be formulated as those of the currently available seasonal influenza quadrivalent vaccines [32–34] and therefore can be administered as a new IAV, IBV, and IDV combination vaccine formulation on an annual basis.

As the world is still grappling with the ongoing COVID-19 pandemic, it is important to keep viruses, such as IDV with zoonotic potentials, in check as some of these viruses have the potential to become human pathogens with deadly consequences.

## Data Availability

No primary data (figures and tables) are included in this article.
